# Nanobody-based CAR-T cells for cancer immunotherapy

**DOI:** 10.1186/s40364-022-00371-7

**Published:** 2022-04-25

**Authors:** Pouya Safarzadeh Kozani, Abdolhossein Naseri, Seyed Mohamad Javad Mirarefin, Faeze Salem, Mojtaba Nikbakht, Sahar Evazi Bakhshi, Pooria Safarzadeh Kozani

**Affiliations:** 1 Department of Medical Biotechnology, Faculty of Paramedicine, Guilan University of Medical Sciences, Rasht, Iran; 2 School of Allied Medical Sciences, Iran University of Medical Sciences, Tehran, Iran; 3 Department of Immunology, Faculty of Medical Sciences, Tarbiat Modares University, Tehran, Iran; 4 Department of Medical Biotechnology, Faculty of Medical Sciences, Tarbiat Modares University, Tehran, Iran; 5 Department of Anatomical Sciences, School of Medicine, Guilan University of Medical Sciences, Rasht, Iran

**Keywords:** Chimeric antigen receptor, Single-chain fragment variable, Nanobody, VHH, Solid tumors, Hematologic malignancy, Cancer immunotherapy, Adoptive cell therapy

## Abstract

Chimeric antigen receptor T-cell (CAR-T) therapy is the result of combining genetic engineering-based cancer immunotherapy with adoptive cell therapy (ACT). CAR-T therapy has been successful in treating various types of hematological cancers. CARs are receptors made of an extracellular domain, a membrane-spanning domain, and an intracellular domain. The extracellular domain of CARs harbors an antigen-targeting domain responsible for recognizing and binding cell surface-expressed target antigens. Conventionally, the single-chain fragment variable (scFv) of a monoclonal antibody (mAb) is used as the antigen-targeting domain of CARs. However, of late, researchers have exploited nanobodies for this aim based on numerous rationales including the small size of nanobodies, their stability, specificity, and high affinity, and their easy and feasible development process. Many findings have confirmed that nanobody-based CAR-Ts can be as functional as scFv-based CAR-Ts in preclinical and clinical settings. In this review, we discuss the advantages and disadvantages of scFvs and nanobodies in regards to their application as the targeting domain of CARs. Ultimately, we discuss various CAR target antigens which have been targeted using nanobody-based CAR-T cells for the treatment of different types of malignancies.

## Introduction

Chimeric antigen receptor T-cells (CAR-Ts) are T lymphocytes that have been genetically engineered to express synthetic CAR molecules on their surface. CAR molecules endow T lymphocytes with the proficiency to recognize cell surface target antigens of interest and mediate exclusive cytotoxicity against cells expressing these antigens [1]. The engagement of CARs with their target antigen triggers downstream activation signaling cascades in T cells in a major histocompatibility complex (MHC)-independent mechanism [1]. In detail, CARs recognize their target antigens via their targeting domain and become activated through the intracellular activation domain [1]. So far, CAR-T therapy has been famous for its ability to mediate remission mostly in patients with relapsed/refractory (R/R) hematological neoplasm such as B-cell acute lymphoblastic leukemia (B-ALL) [2–5]. In 2017, the US Food and Drug Administration (FDA) approved *tisagenlecleucel* for the treatment of patients with B-ALL making it the first CAR-T therapy approved by the FDA for clinical applications [2]. To this date, six CAR-T products have been approved by FDA which include *tisagenlecleucel* for B-ALL and diffuse large B-cell lymphoma (DLBCL), *axicabtagene ciloleucel* for DLBCL and follicular lymphoma (FL), *brexucabtagene autoleucel* for mantle cell lymphoma (MCL), *lisocabtagene maraleucel* for DLBCL, and *idecabtagene vicleucel* and *ciltacabtagene autoleucel* for multiple myeloma (MM) [2, 6–12]. The antigen recognition domain, generally called “targeting domain” of five of these FDA-approved CAR-T products is based on single-chain fragment variable (scFv) of monoclonal antibodies (mAbs) [1]. On the other hand, the targeting domain of *ciltacabtagene autoleucel* is based on single-domain antibodies. In recent years, researchers have focused on other types of targeting domains for CARs. These alternatives include nanobodies, peptides, or ligands [13–17]. In this article, we shine a light on the limitations of scFvs as CAR targeting domains, discuss the advantages of nanobodies as alternative CAR targeting domains, and, ultimately, we review target antigens against which nanobody-based CAR-Ts have been developed and evaluated for the treatment of various types of neoplasms.

### CAR fundamentals

Structurally, CARs are made of several naturally unrelated molecules tailored together as a single chimeric cell surface expressible receptor which is capable of triggering cell activation signals upon encountering the target antigen of interest. The targeting domain of CARs is their most important component in terms of recognizing and interacting with the target antigen of interest. This critical component of CARs is connected to the other parts through a linker (also called *hinge*). The hinge is fused to a transmembrane domain which acts to anchor the whole CAR construct in the host cell membrane and also is a link between the extracellular and the intracellular domains of CARs. The intracellular domain of CARs harbors an activation domain and one or two co-stimulatory domains. The early CARs, named first-generation CARs, did not harbor any co-stimulatory domains [18]. Even though T cells expressing these CARs demonstrated specific antitumor activity towards malignant cells in vitro and in vivo, they mediated poor clinical responses in terms of cytotoxicity and long-term persistence [18–21]. Therefore, these cells were considered clinically non-effective [18–21]. Later, it was revealed that the ineffectiveness of first-generation CAR-Ts for robust clinical persistence could be resolved by incorporating a co-stimulatory domain (for instance, 4-1BB or CD28) into the construct of CARs (between the transmembrane domain and the activation domain) [22–24]. This action was critically essential since target tumor cells do not generally express a co-stimulatory receptor ligand on their surface [25]. T cells genetically engineered to express these CARs were named second- and third-generation CAR-Ts with second-generation CARs having one co-stimulatory domain and third-generation CARs having two co-stimulatory domains [26–28]. Second- and third-generation CAR-Ts demonstrated improved T-cell activation, enhanced in vitro expansion upon target antigen engagement, more durable in vivo persistence, and superior tumoricidal capacity [26–28]. It is worth mentioning that all of the CAR-T products approved by the US FDA are second-generation CAR-Ts [6–11, 29]; despite the fact that third-generation CAR-Ts have demonstrated improved proliferation and persistence in vivo [30].

The co-stimulatory domain of CARs has various effects on the metabolism and fate of the CAR-expressing T cells. In this regard, Kawalekar et al. demonstrated that the 4-1BB co-stimulatory domain in the construct of CARs mediates T-cell central memory phenotype development [31]. Moreover, it also mediates improved mitochondrial biogenesis and oxidative breakdown of fatty acids leading to enhanced T-cell expansion, activity, and persistence [31]. On the other hand, CD28 co-stimulatory domain mediates effector memory cell phenotype development and improves the glycolysis process in T cells [31]. Moreover, another study has reported that the 4-1BB co-stimulatory domain alleviates T-cell exhaustion mediated by scFv-induced CAR aggregation and tonic signaling [32]. On the contrary, in regards to the CD28 co-stimulatory domain, one study has reported that target antigen-independent signaling contributes to CD28-harboring CAR-T exhaustion in vivo [33]*.* Of note, various studies have indicated that 4-1BB can counteract anergy during chronic viral infections [32, 34, 35]. Additionally, preclinical data imply that cytokine release is often superior with CAR-Ts possessing the CD28 co-stimulatory domain in compassion with CAR-Ts with the 4-1BB co-stimulatory domain [36]. These findings demonstrate that CAR-Ts expressing 4-1BB or CD28 co-stimulatory domains may perform in different ways following in vivo administration, proposing a deliberate design for future CAR-T products based on the expected clinical outcomes.

**Fig. 1:**
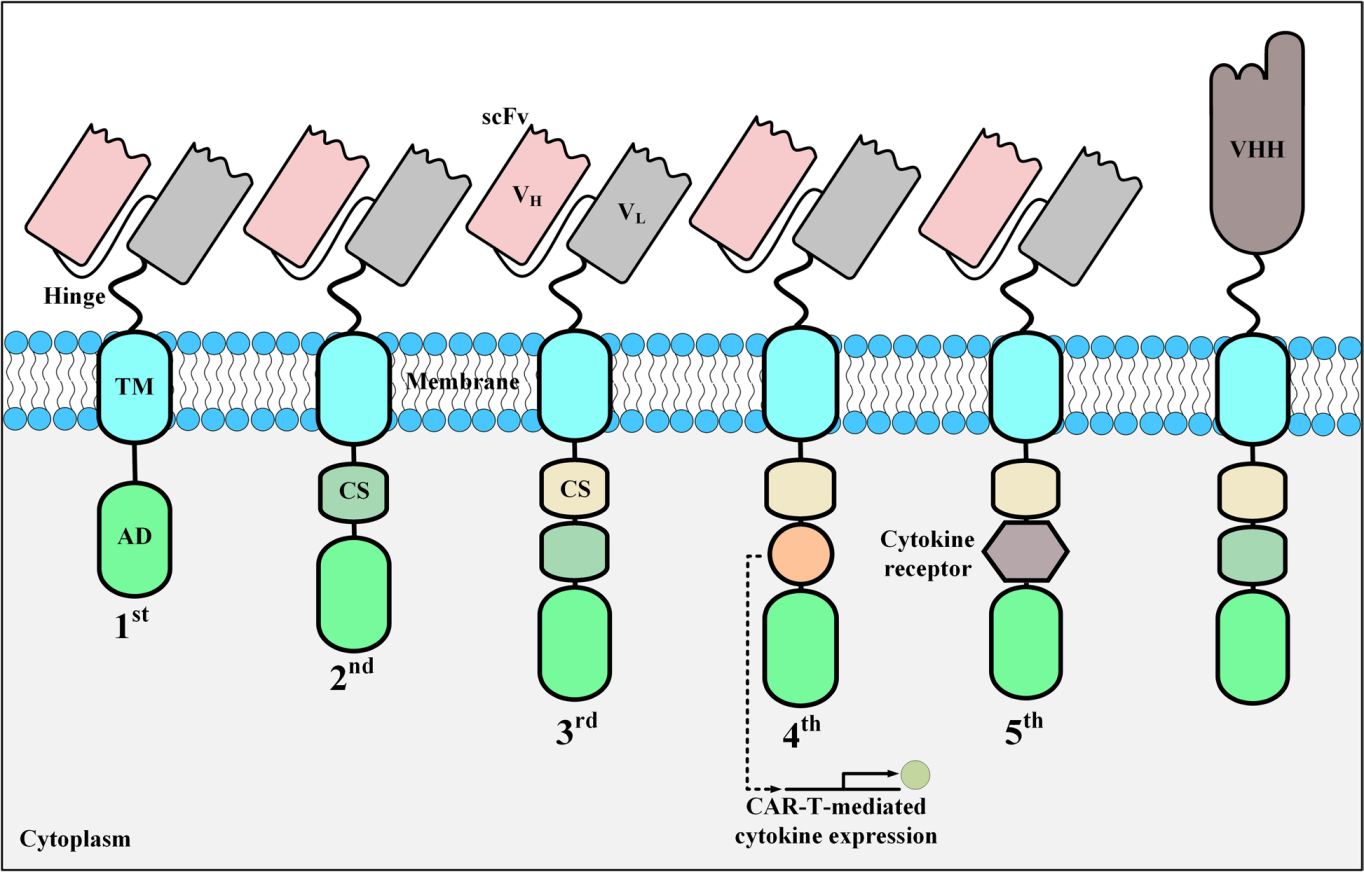
The structural characteristics of CAR generations developed to date. These five generations of CARs only have differences in their intracellular domains. AD, activation domain; CS, costimulatory domain; scFv, single-chain fragment variable; TM, transmembrane domain; V_H_, heavy chain variable domain; VHH, single variable domain on a heavy chain; V_L_, light chain variable domain

Further genetic manipulation of CAR constructs aims at endowing CAR-Ts with the ability to secrete a cytokine of interest for enhancing their antitumor activity for the treatment of solid tumors [37, 38]. Tumor site delivery of a cytokine of interest by CAR-Ts is believed to have significant modulating effects on the tumor microenvironment (TME) of solid tumors [37]. Therefore, fourth-generation CAR-Ts merge the direct tumoricidal functionality of CAR-Ts with the immune-modulating abilities of the tumor-site delivered cytokines without the adverse effects of the systemic administration of such cytokines [37]. Moreover, fifth-generation CAR-Ts are second-generation-based CARs that harbor an intracellular receptor of a cytokine of interest on their intracellular domain [19, 39]. Different CAR generations have been illustrated in Fig. 1.

### scFvs and VHHs as the targeting domain of CARs

**Fig. 2:**
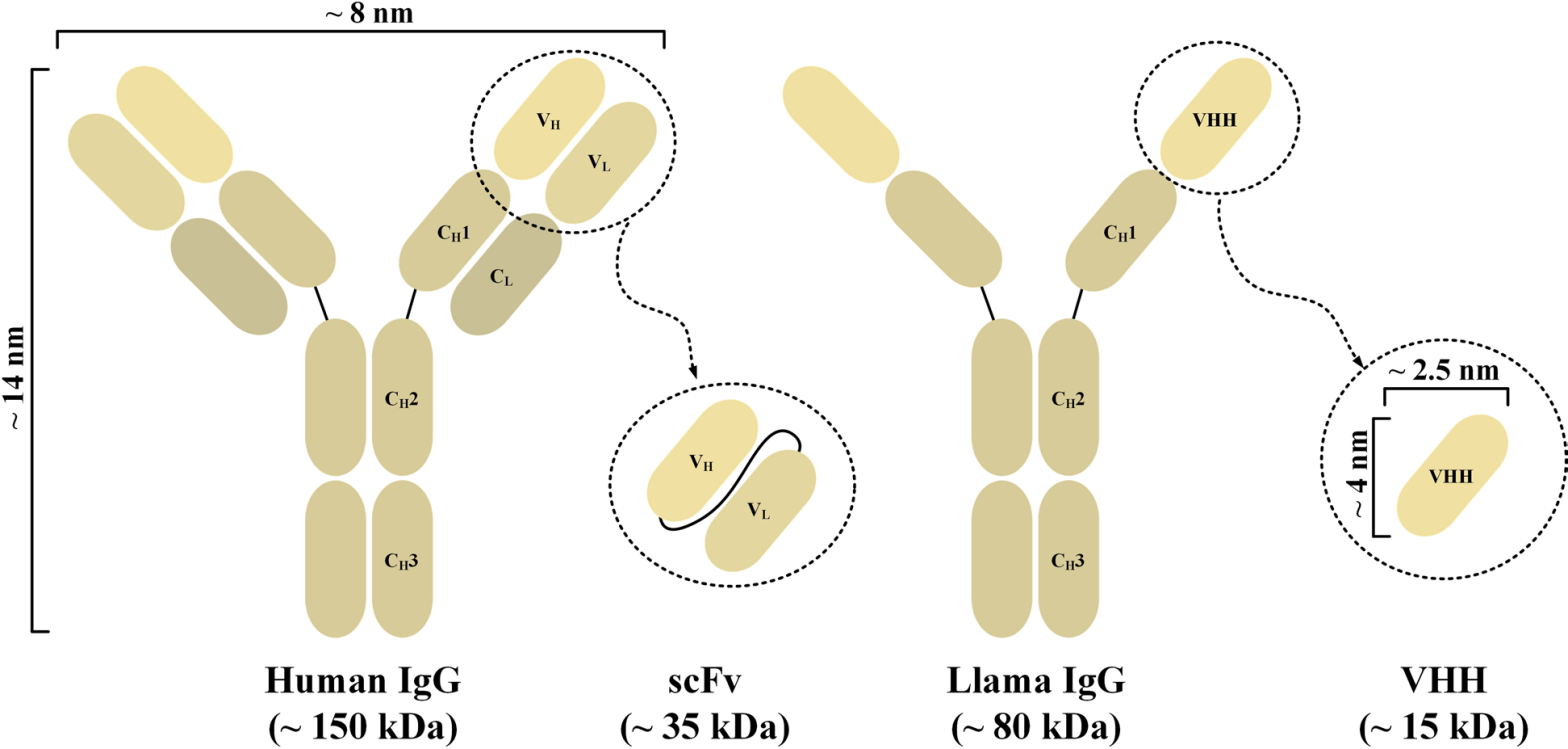
An illustration of a human and a llama immunoglobulin G and their applicable derivatives as CAR targeting domains. C_H_1, heavy chain constant domain 1; C_H_2, heavy chain constant domain 2; C_H_3, heavy chain constant domain 3; C_L_, light chain constant domain; IgG, immunoglobulin G; V_H_, heavy chain variable domain; VHH, single variable domain on a heavy chain; V_L_, light chain variable domain

The targeting domain of CAR-Ts is mostly based on the scFv of a mAb. scFvs are broadly applied as CAR targeting domains and for the development of T-cell-redirecting bispecific antibodies (TRBA) owing to their compact size and high affinity and specificity [40]. Moreover, single variable domain on a heavy chain (VHH), also known as nanobodies, have also been used as the targeting domain of CARs. Nanobodies are derived from the variable domain of heavy chain-only antibodies (HcAbs) (Fig. 2) [41, 42]. Naturally, animals from the *Camelidae* family as well as sharks produce HcAbs [41, 42]. Nanobodies recognize and bind their target antigen with similar binding ability and specificity in comparison with those of traditional full-length mAbs or scFvs [41]. Moreover, the solubility and stability of nanobodies are also comparable to those of full-length mAbs [41]. Of note, nanobodies possess these characteristics even in the absence of the variable light-chain (V_L_) and constant domains [41].

The major idea behind the application of nanobodies as the targeting domain of CARs, as an alternative to scFvs, emerged due to various limitations concerning the application of scFvs. For example, a linker is utilized to fuse the variable heavy-chain (V_H_) and V_L_ domains to produce an scFv [43]. Following CAR-T infusion, the host immune system can mediate immune reactions against such linkers through the formation of neutralizing antibodies due to the immunogenicity of the linkers [44, 45]. On the contrary, in the case of VHHs, the risk of immunogenicity is less likely since nanobodies lack such synthetic linker peptides.

Moreover, the framework of antibodies from murine sources is also known to be acting as a source of immunogenicity [44, 45]. The formation of neutralizing antibodies against the scFvs of CAR-Ts after infusion can remarkably restrict the tumoricidal functionality of the infused CAR-Ts [44, 45]. Generally, a great percentage of clinically applied mAbs are of murine origin; therefore, they are capable of inducing the formation of *human anti-mouse antibodies* (*HAMAs*) upon administration to humans [46, 47]. HAMAs can significantly impair the clinical effectivity of mouse-based mAbs [46, 47]. For instance, Gruber et al. reported the formation of HAMAs in colorectal cancer patients under treatment with the mAb *CO17-1A* [48]. However, these researchers signified that such anti-idiotypic reactions had no substantial effects on the clinical outcomes [48]. Another study by Herlyn et al. also reported the formation of anti-idiotypic antibodies against the mAb *CO17-1A* in patients with different types of carcinomas [49]. Moreover, anti-idiotypic immune responses have been reported against scFvs when used as the targeting domain of CARs. In this regard, Lamers et al. have reported the emergence of humoral and cellular immune responses against the targeting domain of autologous carbonic anhydrase IX (CAIX)-redirected CAR-Ts in patients with renal cell carcinoma [50]. According to this report, such immune responses resulted in restricted peripheral persistence of adoptively transferred CAIX-redirected CAR-Ts [50]. In detail, these anti-idiotypic humoral immune responses considerably neutralized the tumoricidal functionality of the mentioned CAR-Ts [50]. Moreover, the developed HLA-mediated cellular immune responses were against the complementarity-determining regions (CDRs) and framework regions of the CAR scFv [50].

So far, there have not been any reports on the formation of neutralizing antibodies against nanobodies when used as the targeting domain of CAR-Ts following their infusion into human subjects. However, Ackaert et al. conducted a study to investigate the possible immunogenicity of two nanobodies that are currently being investigated in Phase II clinical trials [51]. Of note, one of the nanobodies was against HER2 and the other one was specific for macrophage mannose receptor utilized for nanobody-based imaging of breast cancer and tumor-associated macrophages, respectively. Based on the data collected from 20 patients, the researchers concluded that nanobodies are poorly immunogenic which might support their further application as targeting moieties [51]. As a proposed strategy for this low immunogenicity level, one study has implied that the sequence of nanobodies is much more similar to the human V_H_, making nanobodies more compatible and less immunogenic for numerous clinical applications [41]. However, antibody humanization is considered a stratagem for alleviating the possibility of anti-idiotypic immune reactions against non-human antibodies [52, 53]. In this approach, the framework regions of a given murine scFv are substituted with similar human framework regions (or the CDRs of a given murine scFv are grafted onto the framework regions of a similar human mAb) [52, 53]. Moreover, nanobodies also tend to have advantages over scFvs in the context of humanization since the humanization process of nanobodies is believed to be much easier and time-consuming, mainly because of the fewer residue substitutions performed in this process [53]. CAR-Ts with humanized nanobodies or scFvs as their targeting domains (called humanized CAR-Ts) have been extensively evaluated in numerous clinical studies [5, 54–60].

**Fig. 3:**
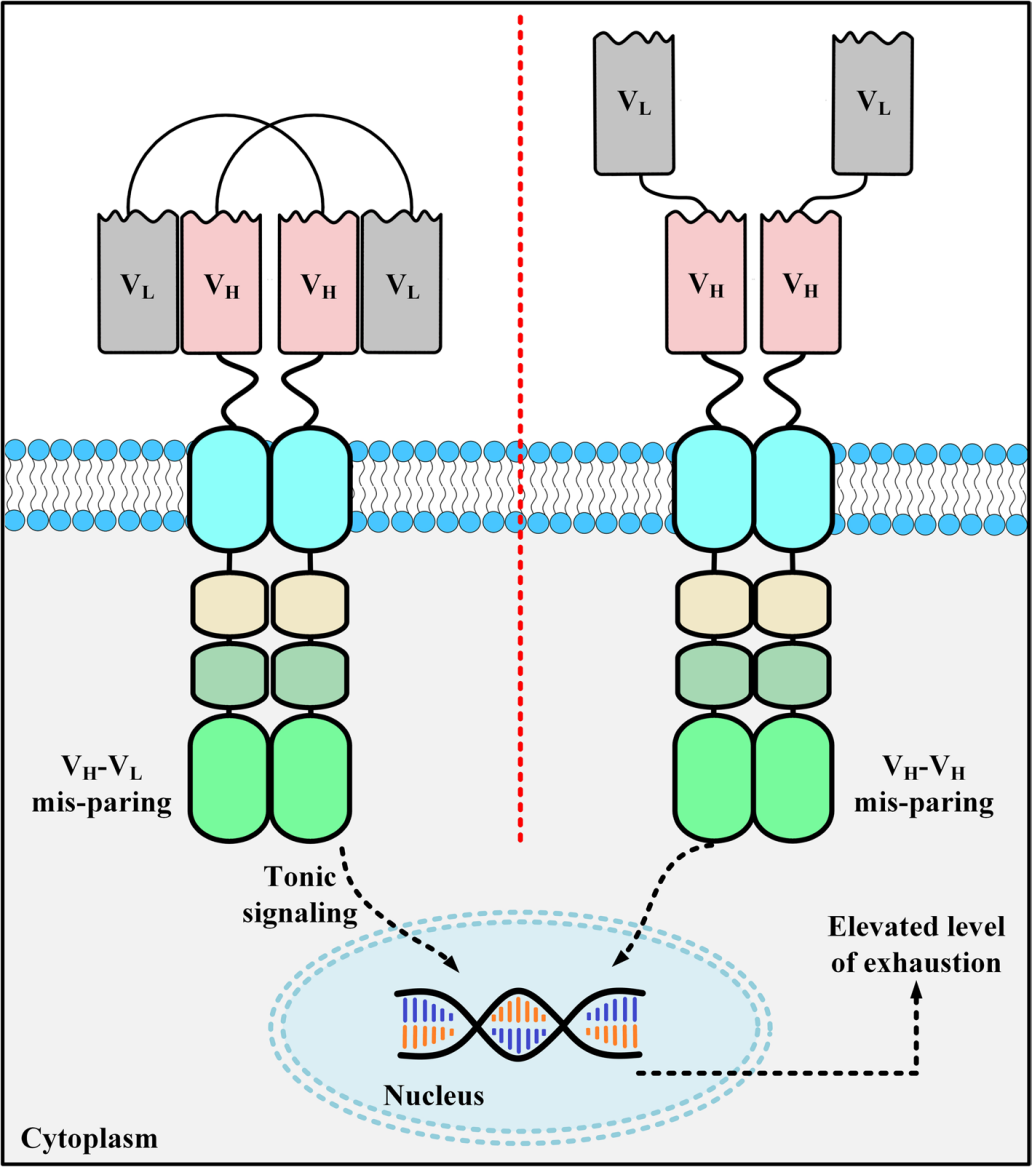
scFv aggregation on the surface of CAR-Ts. There are two action mechanisms following which scFv aggregation occurs on the surface of CAR-Ts. The first mechanism (left panel) is called V_H_-V_L_ mispairing and involves the paring between the V_L_ domain from one CAR with the V_H_ domain of another CAR. The second mechanism (right panel) is termed V_H_-V_H_ pairing and happens when the V_H_ domain of a CAR pairs with a V_H_ domain from another CAR. V_H_, heavy chain variable domain; V_L_, light chain variable domain

Another limitation of utilizing scFvs as CAR targeting domains is CAR aggregation leading to CAR-T exhaustion [32, 44, 61]. This occurrence takes place independent of target antigen engagement [32, 44, 61]. Researchers have suggested that this occurrence is possibly originated from the variable domains of CAR scFvs [32]. Other studies have also added that the high tendency of scFvs for self-aggregation is mostly derived from the freely exposed hydrophobic residues on their variable domains or the poor V_H_ or V_L_ domain folding stabilities [61–64]. CAR aggregation on the surface of CAR-Ts triggers the activation and cytotoxic signaling cascades of the effector cells which can lead to T-cell exhaustion (Fig. 3) [65]. On the contrary, nanobody-based CAR-Ts do not tend to have the limitations of CAR surface aggregation and target antigen-independent effector cell activation. This advantage of nanobodies is one of the main reasons for investigating them as an alternative to scFvs for CAR targeting domains.

**Fig. 4:**
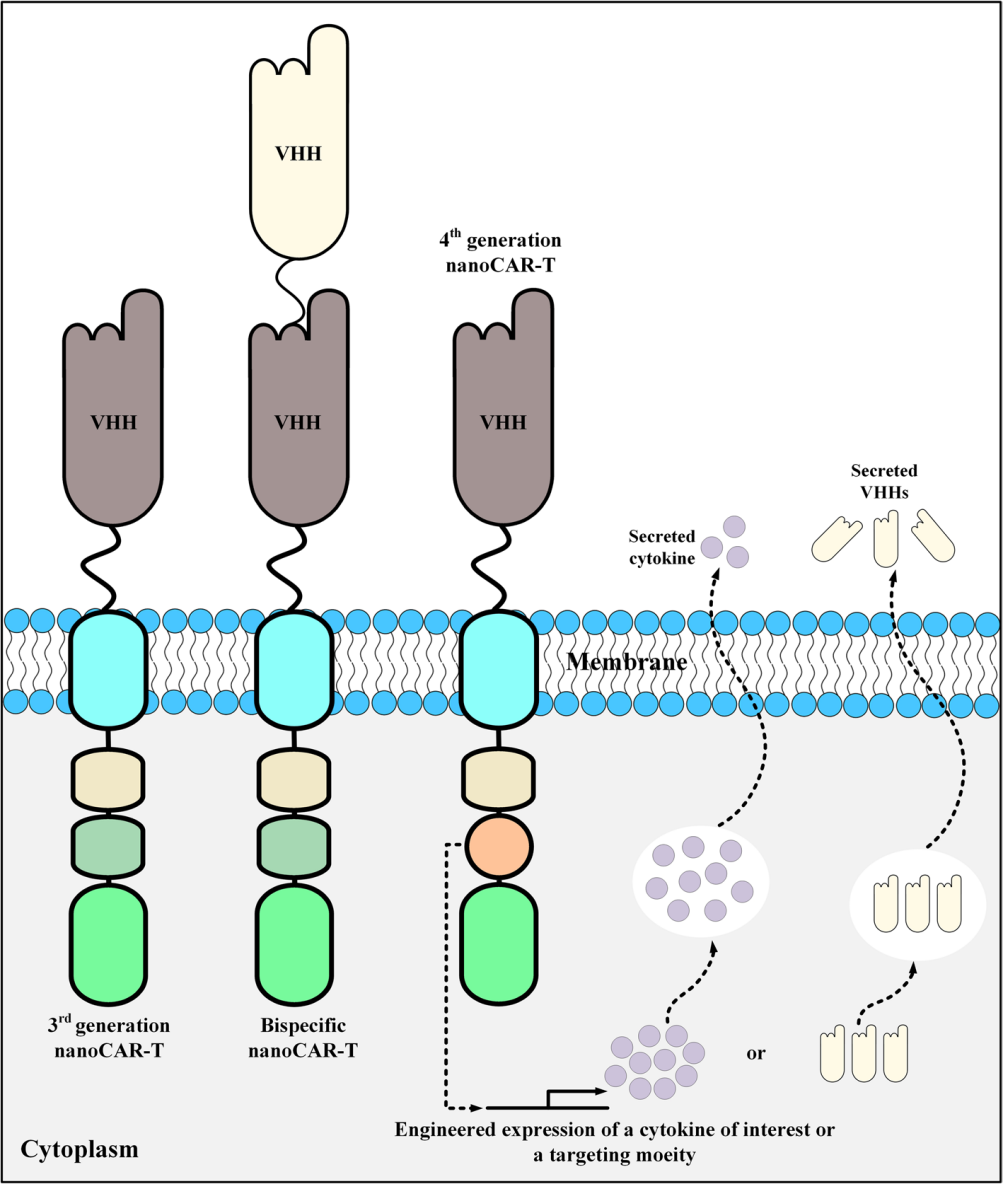
Various types of VHH-based CAR-Ts. Bispecific VHH-based CAR-Ts have a targeting domain made by fusing two VHHs using a linker. Moreover, fourth-generation VHH-based CAR-Ts are CAR-Ts genetically manipulated to secrete a cytokine of interest or a particular type of targeting moiety (such as VHHs). nanoCAR-T, VHH-based CAR-T; VHH, single variable domain on a heavy chain

Another CAR-T-related field in which scFvs tend to have limitations is the generation of tandem CARs (TanCARs) [66]. TanCARs are bispecific CARs that are generated from two tandem antigen-binding domains specific for two distinct target antigens or two distinct epitopes of a particular target antigen [66]. For such applications, VHHs seem to be much more favorable targeting moieties as compared with scFvs. Additionally, researchers have demonstrated that V_H_ and V_L_ domains of two separate scFvs may unintentionally develop crossed pairs leading to affinity loss [66]. Furthermore, insertion of the large DNA fragments of scFvs into retroviral vectors might contribute to lowering the efficiency of transfection and viral packaging [67–69]. Another advantage of nanobodies over scFvs is their long CDR3 which enables them to bind particular epitopes that are out of reach of conventional mAbs [70–72]. All of these advantages of VHHs have encouraged investigators to employ them as CAR targeting domains (Fig. 4). In the upcoming section, we will highlight target antigens against which nanobody-based CAR-T have been developed and investigated in preclinical and clinical studies.

### Target antigens against which nanobody-based CAR-T cells have been developed

#### Vascular endothelial growth factor receptor 2 (VEGFR2)

VEGFR2 is a receptor of vascular endothelial growth factor (VEGF) on the surface of endothelial cells [73]. This receptor plays roles in angiogenesis regulation and tumorigenesis [73]. VEGFR2 overexpression has been documented in various types of cancers such as head and neck squamous cell carcinomas (HNSCCs) [73]. VEGFR2 has been studied as a target for CAR-T therapy in recent years [74]. In terms of nanobody-based CAR-Ts, in 2019, Hajari Taheri et al. generated second-generation VHH-based VEGFR2-redirected CAR-Ts via CAR-encoding plasmid electroporation, and reported that these cells expressed CD69 and CD25 activation markers on their surface upon co-cultivation with VEGFR2-expressing target cells [75]. Moreover, the researchers added that these effector cells also demonstrated target antigen-dependent IL-2 and IFN-γ production and secretion [75]. These CAR-Ts also mediated specific tumoricidal activity against human VEGFR2-expressing 293-KDR cells [75]. Conclusively, these researchers suggested the potential of these VHH-based CAR-Ts for VEGFR2 targeting overexpressed on tumor vasculatures [75]. However, more comprehensive studies can further validate these findings since, to our knowledge, this is the only report on using VHH-based CAR-Ts for targeting VEGFR-2.

#### Human epidermal growth factor receptor 2 (HER2)

HER2 is a member of the epidermal growth factor receptor family with tyrosine kinase activity [76]. This surface antigen has critical roles in signaling pathways mediating cell proliferation and tumorigenesis in various types of malignancies [76]. HER2 overexpression has been reported in various neoplasms such as breast and gastric cancers making it a suitable target for prognostic aims as well as CAR-T therapy [76]. HER2 has been broadly investigated as a CAR-T target, especially in recent years [77, 78]. Jamnani et al. genetically manipulated Jurkat cells to express VHH-based HER2-redirected CARs [79]. In detail, these oligoclonal CAR-Ts were generated using five VHH clones, as a set of oligoclonal HER2-targeting nanobodies, fused to CD28-CD3ζ and CD28-OX40-CD3ζ signaling domains [79]. Jamnani et al. reported that oligoclonal CAR-Ts demonstrated enhanced expansion, cytokine secretion, and antitumor activity in comparison with those of CAR-Ts generated using each individual VHH in vitro [79]. They also added that coupling the enhanced targeting ability of oligoclonal VHHs with third-generation CARs can remarkably improve the tumoricidal activity of engineered T cells [79]. However, the findings of this study should be carefully interpreted since the Jurkat T lymphocyte cell line has been utilized as the effector cells for the expression of CARs. Moreover, preclinical data are highly required to be able to draw conclusions on the efficacy and safety profile of nanobody-based CAR-Ts for targeting HER2.

#### Tumor-associated glycoprotein 72 (TAG-72)

TAG-72 is a membrane-spanning antigen with mucin-like characteristics [80]. The overexpression of TAG-72 has been observed in a variety of malignancies such as pancreatic, breast, colorectal, and ovarian cancers [80]. TAG-72 has been targeted using scFv-based CAR-Ts in a wide range of malignancies [16]. However, nanobody-based TAG-72-redirected CAR-Ts are not majorly investigated. In 2013, Sharifzadeh et al. generated CAR-expressing oligoclonal T cells harboring anti-TAG-72 nanobodies based on the fact that natural oligoclonal T cells mediate more efficacious antitumor responses in cancer patients in comparison with those of single monoclonal T cells [81]. These researchers hypothesized that the application of these CAR-Ts can result in reduced immunogenicity and targeting multiple sites on a single tumor cell [81]. According to this report, supraphysiological concentrations of soluble TAG-72 antigen did not interfere with the antitumor activity of these CAR-Ts [81]. Moreover, stimulation of these CAR-Ts with TAG-72-expressing cell lines such as LS-174 T and MCF7 resulted in their target significant antigen-dependent proliferation [81]. These CAR-Ts also mediated IL-2 production and secretion and specific cytotoxicity upon target tumor cell engagement [81]. Conclusively, these researchers suggested that this approach can reverse multiple tumor immune evasion mechanisms and prevent CAR immunogenicity [81]. However, since these findings does not include preclinical as well as clinical data, it cannot be concluded that this approach might be effective in the reversion of tumor immune evasion mechanisms. For such deductions, more in-depth information is required.

#### Prostate-specific membrane antigen (PSMA)

PSMA is a type II cell surface-expressed antigen present in all forms of prostate tissue including carcinoma [82]. Over the past years, PSMA has been used as a diagnostic and therapeutic target in prostate cancer [82]. PSMA has been known as a promising target for scFv-based CAR-T therapy of local and advanced prostate cancer [83]. Nanobody-based CAR-Ts have also been generated for targeting this antigen. In 2019, researchers generated VHH-based PSMA-redirected CAR-Ts using a PSMA-targeting nanobody named *NBP* [84]. In detail, it was reported that these CAR-Ts demonstrated significant target antigen-dependent expansion, cytokine secretion, and CD69 activation marker upregulation upon co-cultivation with PSMA-expressing LNCaP cells [84]. Even though these researchers suggested that these findings demonstrate the potential of VHH-based CAR-Ts for CAR-T therapy of prostate cancer, broader investigations including preclinical assessments are required for such conclusions since the mentioned study only includes in vitro assessments [84]. In 2020, Hassani et al. reported the findings of a similar study assessing the antitumor activity of Jurkat cells engineered to express VHH-based PSMA-redirected CARs [85]. According to this study, these CAR-Ts mediated PSMA-triggered antitumor activity and IL-2 secretion, and upregulated the surface expression of CD25 activation marker upon co-culturing with LNCaP cells [85]. However, it is worth mentioning that the findings of this study using Jurkat cells cannot imply that similar results can be obtained while using primary T lymphocytes, as the CAR-expressing effector cells. Same as the previous report on the VHH-based PSMA-redirected CAR-Ts, this study also reported in vitro evaluations which does not guarantee the applicability and efficacy of this platform in preclinical and clinical settings.

#### Glypican 2 (GPC2)

GPC2 is a transmembrane heparan sulfate proteoglycan with critical roles in neuronal cell adhesion [86]. Li et al. have reported that GPC2 overexpression is observed in about 50% of neuroblastoma cases correlating with poor overall survival of the patients [87]. These researchers also reported that CRISPR/Cas9- or siRNA-mediated inhibition of GPC2 expression suppresses the outgrowth of neuroblastoma tumor cells [87]. Li et al. also isolated nanobodies specific for GPC2 using *phage display technology,* and demonstrated that these nanobodies mediate the inhibition of active β-catenin signaling by interrupting the interaction between GPC2 and Wnt3a [87]. These researchers used the isolated nanobodies for the generation of immunotoxins and CARs [87]. In detail, GPC2-redirected immunotoxins suppressed neuroblastoma growth, and consistent with this finding, VHH-based GPC2-redicted CAR-Ts also demonstrated significant antitumor activity against IMR5 cells with high levels of GPC2 expression [87]. Exposure of these CAR-Ts to IMR5 cells also resulted in significant production and secretion of IFN-γ and TNF-α as compared with control CAR-Ts [87]. Furthermore, these CAR-Ts significantly controlled the growth of metastatic tumors and reduced tumor burden in preclinical mouse models engrafted with IMR5 cells [87]. Conclusively, based on these findings, Li and co-workers proposed GPC2 as a promising target and added that GPC2 targeting via nanobody-based immunotherapeutics might be favorable for neuroblastoma treatment [87]. Of note, more preclinical data can further validate the findings of this study while paving the way for the evaluation of nanobody-based GPC2-redirected CAR-Ts in early phase clinical settings.

#### CD38

CD38 is a cell-surface expressed glycoprotein expressed on plasma cells and various lymphoid and myeloid cell populations [88]. The uniform and high-level expression of this surface marker have rendered it a suitable target for targeted cancer therapies [88]. Such cancer therapies include mAb-based therapies (using CD38-specific mAbs such as *daratumumab* and *isatuximab*), adoptive cell therapy (ACT) using CAR-Ts redirected against CD38, and radioimmunotherapy [88]. CAR-T-mediated CD38 targeting has been mostly studied using scFv-based CAR-Ts for the treatment of MM [89]. In 2018, An et al. developed a novel CD38-specific nanobody and used it as the targeting domain of CARs to generate VHH-based CD38-redirected CAR-Ts [90]. These researchers reported that their VHH-based CAR-Ts demonstrated significant antitumor functionality, proliferation, and IL-2, IFN-γ, and TNF-α secretion upon encountering CD38-expressing cell lines (including LP-1, RPMI 8226, OPM2, and MOLP8) and primary patient-derived MM cells [90]. It was also demonstrated that these CAR-Ts do not mediate antitumor activity against CD38-knocked out LP-1 cells or CD38-deficient K562 cells [90]. It is worth mentioning that these researchers also reported that these CAR-Ts mediated minor cytotoxicity against CD38-expressing T cells, B cells, and natural killer (NK) cells [90]. Moreover, these CAR-Ts induced efficient tumor growth suppression in mouse preclinical models established using RPMI 8226 cells [90]. Taken together, An et al. proposed that VHH-based CD38-redirected CAR-Ts can be a reliable approach for the treatment of patients with MM [90]. However, profound preclinical and clinical data are required to safely conclude that CD38 targeting via nanobody-based CAR-Ts does not mediate off-tumor cytotoxicity against T cells, B cells, and NK cells proficient in the expression of CD38. Moreover, more in-depth preclinical and clinical assessments are warranted to safely claim that nanobody-based CD38-redirected CAR-Ts can be a feasible approach for the treatment of patients with MM.

#### CD33

CD33 is a myeloid differentiation cell surface-expressed antigen present on acute myeloid leukemia (AML) blasts of a high percentage of patients [91]. This surface marker has been used as a target for antibody-based therapeutics [91]. However, the low level of CD33 expression alongside its slow internalization restricts antibody-dependent cell-mediated cytotoxicity (ADCC) and drug accumulation [91]. In this regard, CD33 has also been used as a CAR-T target for various types of cancers [92]. In 2020, De Munter et al. used a CD33-specific nanobody (generated after the immunization of llamas against the extracellular domain of CD33 using soluble proteins) to generate VHH-based CD33-redirected CAR-Ts [93]. These researchers confirmed the expression of CD33 on a range of AML cell lines including U937, HL60, MOLM13, and Thp1 [93]. The CAR-Ts generated using this nanobody demonstrated specific target cell lysis and cytokine secretion upon co-cultivation with target AML cell lines [93]. The researchers also indicated that VHH-based CD33-redirected CAR-Ts with the 4-1BB co-stimulatory domain demonstrated enhanced antitumor performance in comparison with VHH-based CD33-redirected CAR-Ts with the CD28 co-stimulatory domain [93]. Furthermore, in vivo assessments demonstrated that these CAR-Ts were capable of mediating prolonged survival in preclinical mouse models inoculated with the CD33-expressing Thp1 cell line [93]. However, this study also reported the cytotoxicity of VHH-based CD33-redirected CAR-Ts against CD34-expressing hematopoietic precursor cells (HPC) [93]. Conclusively, De Munter et al. indicated that nanobodies have various advantages over scFvs; for instance, they do not aggregate on the T cell surface, which prevents premature T-cell activation and exhaustion, and they are unlikely to lose affinity [93]. Of note, in regards to the reported off-tumor cytotoxicity of VHH-based CD33-redirected CAR-Ts against CD34^+^ HPC, careful preclinical and clinical investigations should be taken into consideration since such toxicities can result in serious clinical complications in CAR-T recipients.

#### CD7

CD7 is a cell surface-expressed glycoprotein with normal expression restricted to NK cells and T lymphocytes [94]. A great proportion of T-cell acute lymphoblastic leukemia (T-ALL) and T-cell lymphomas exhibit CD7 overexpression [95, 96]. Therefore, CD7 has been considered as a target for various types of immunotherapy (especially for the treatment of various kinds of T-cell malignancies). For instance, the application of immunotoxins redirected towards CD7 has been investigated for the treatment of T-cell leukemias and lymphomas [97, 98]. Likewise, CD7-redirected CAR-Ts have been extensively studied for the treatment of T-cell malignancies [99]. 

In 2021, Zhang et al. reported the findings of a Phase I clinical trial (NCT04004637) investigating the safety and efficacy of autologous fratricide-resistant nanobody-based CD7-redirected CAR-T cells [100]. Fratricide is described as self-targeting of CAR-Ts which is resulted from the expression of the CAR-T target antigen on CAR-expressing T cells [99]. This phenomenon significantly reduces CAR-T in vivo persistence and tumoricidal activity [99]. According to Zhang et al., 8 patients were enrolled in this clinical trial, 5 of which had R/R T-cell acute lymphoblastic leukemia/lymphoma (ALL/LBL), and 3 had R/R early T-cell precursor (ETP)-ALL/LBL [100]. The reported overall response rate at one month was 100% while the complete remission (CR) rate at three months was 75% [100]. Two patients (25%) experienced grade 2 cytokine release syndrome (CRS) while the other patients (75%) demonstrated only grade 1 CRS [100]. Case 2 experienced an abdominal infection leading to the death of the patient at month 3, while the patient was still in minimal residual disease (MRD)^–^ condition [100]. Only two patients relapsed, one of which was case 3 who was MRD^–^ for seven months but appeared MRD^+^ in the bone marrow at month 8 [100]. However, this patient underwent CAR-T therapy again and regained CR. The other patient with relapsed disease was case 7 who demonstrated disease relapse with CD7^–^ leukemic blasts at month 6 rendering CD7-redirected CAR-T therapy ineffective for targeting and eradicating malignant cells [100]. Conclusively, these researchers suggested that autologous VHH-based CD7-redirected CAR-Ts are well-tolerated and may provide a significant therapeutic capability for the treatment of patients with CD7^+^ T-cell malignancies [100]. Clinical trials with broader patient populations may provide new insights into the safety and efficacy of these nanobody-based CAR-Ts.

The safety and efficacy of allogeneic CD7-redirected CAR-Ts have been investigated in another clinical trial involving patients with R/R T-cell leukemia/lymphoma [101]. Pan et al. have published a report of the results of this Phase I trial [101]. According to this report, among 20 patients that were administrated with these CAR-Ts, 18 patients (90%) demonstrated grade 1–2 CRS whereas 2 patients (10%) experienced grade 3–4 CRS [101]. Other documented toxicities included grade 3–4 cytopenia in all of the patients (100%), grade 1–2 GvHD in 12 patients (60%), and grade 1–2 neurologic toxicity in 3 patients (15%) [101]. It is worth mentioning that all of the mentioned adverse effects were both controllable and reversible except for one patient who died due to pulmonary hemorrhage [101]. In terms of effectiveness, 18 patients (90%) accomplished CR and 15 patients were still in remission at the median follow-up of 6.3 months [101]. Conclusively, these data exhibited that allogeneic CD7-redirected CAR-Ts mediated a satisfactory CR rate and were well-tolerated in individuals with T-cell neoplasms [101]. Such findings accentuate the fact that careful clinical care needs to be taken into consideration to take all CAR-T therapy adverse events under control and to avoid mortality as much as possible.

#### Mucin 1 (MUC1)

MUC1 is a heterodimeric surface protein aberrantly overexpressed in more than 90% of breast cancers [102]. The dysregulated expression of MUC1 in breast neoplasms is based on genetic modifications and transcription dysregulations [102]. These characteristics of MUC1 have made it a great target for cancer immunotherapy. In 2009, Bakhtiari et al. generated CAR-expressing Jurkat cells equipped with anti-MUC1 nanobodies and reported that these cells could target MUC1-expressing MCF7 breast cancer cells [103]. These researchers proposed that nanobody-based MUC1-redirected CAR-Ts may have effective and exclusive tumoricidal capabilities and are non-immunogenic; therefore, they can be counted on as suitable candidates for clinical applications [103]. In 2011, Iri-Sofla et al. generated second-generation nanobody-based MUC1-redirected CAR-Ts (using Jurkat cells as the effector cells) and evaluated the applicability of the PhiC31 integrase system for optimizing CAR transgene transduction and expression efficiency [104]. These researchers reported that PhiC31 integrase can be efficiently used for stable transduction of the Jurkat cell line [104]. Moreover, in 2012, Khaleghi et al. developed nanobody-based MUC1-redirected CAR-Ts equipped with the OX40 co-stimulatory signaling domain and caspase 8-based suicide switches [105]. These researchers reported that these CAR-Ts mediated target antigen-dependent IL-2 secretion after stimulation by MUC1-expressing tumor cell lines [105]. They also reported more than 90% decrease in the number of CAR-Ts, 24 h following the addition of dimerizing agents [105]. In 2021, Rajabzadeh et al. generated second-generation nanobody-based MUC1-redirected CAR-Ts using camelid-derived anti-MUC1 nanobodies and reported that these cells demonstrated target antigen-dependent IL-2, TNFα, and IFN-γ secretion and antitumor activity against MUC1-expressing cancer cell lines including T47D and MCF-7 [102]. Such data might support the tumoricidal capability of MUC1-redirected nanobody-based CAR-Ts; however, in-depth in vivo studies using preclinical mouse models alongside careful clinical investigations still need to be taken into consideration for demonstrating the efficacy and safety of nanobody-based MUC1-redirected CAR-Ts for the selective elimination of MUC1^+^ malignant cells.

#### Epidermal growth factor receptor (EGFR)

**Fig. 5:**
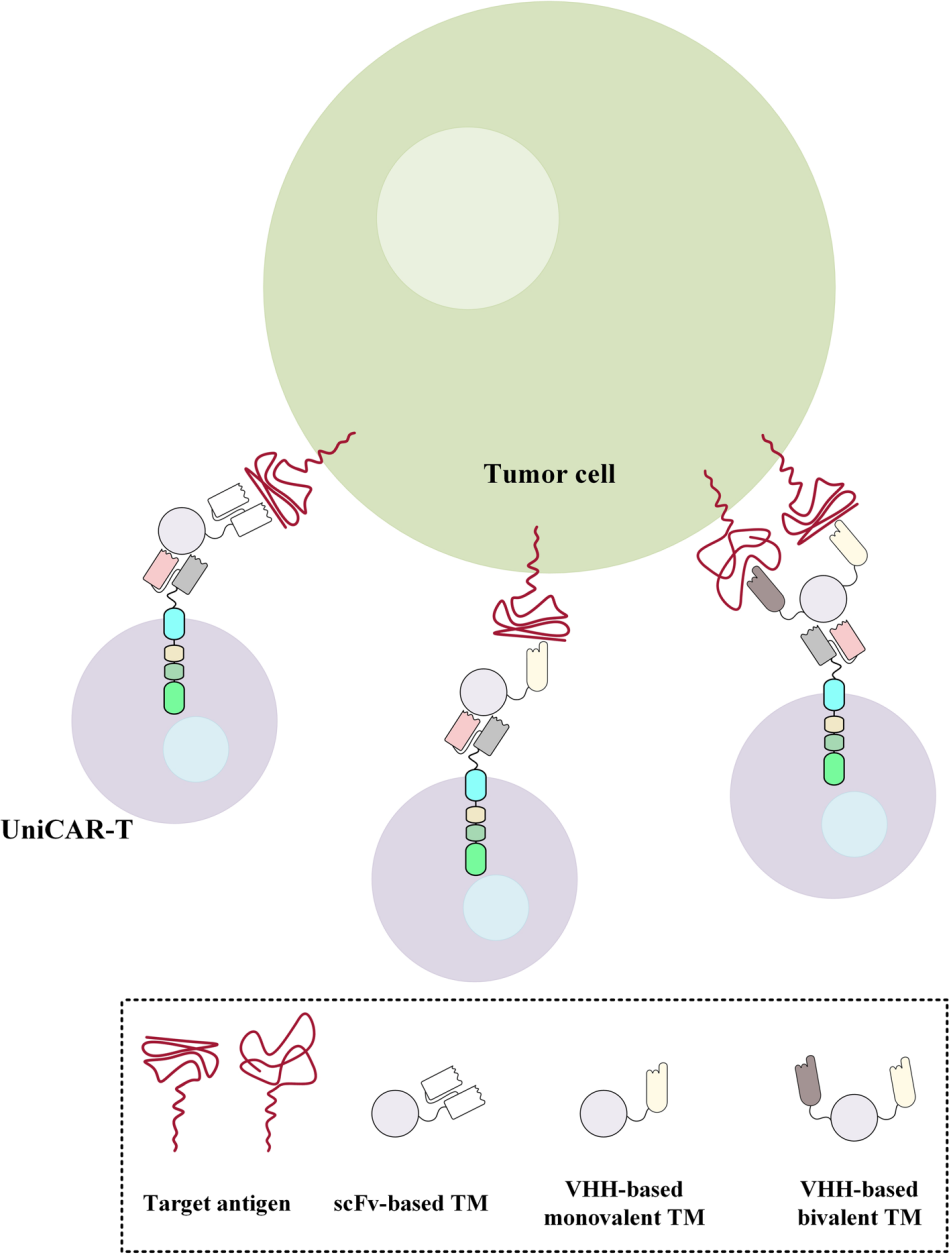
Redirection of UniCAR-expressing T cells using scFv-based, monovalent VHH-based, and bivalent VHH-based targeting modules targeting a particular target antigen. The UniCARs expressed on the surface of effector T cells are responsible for the signal transduction process and effector cell activation. The targeting modules only redirect UniCAR-expressing T cells against a particular cell surface antigen. As proposed by Albert et al., bivalent VHH-based targeting modules are capable of redirecting UniCAR-expressing T cells towards cancer cells expressing low levels of a target antigen (in this case, EGFR) [109]. scFv, single-chain fragment variable; TM, targeting module; UniCAR-T, UniCAR-expressing T cell; VHH, single variable domain on a heavy chain

EGFR is a receptor tyrosine kinase overexpressed in various types of cancers including breast, head and neck, and prostate cancers [106]. Conventional scFv-based EGFR-redirected CAR-Ts have been investigated for targeting various types of cancers [107]. However, VHH-based CAR-Ts targeting this tumor-associated antigen (TAA) have not been broadly investigated. In this regard, Albert et al. generated nanobody-based EGFR-redirected targeting modules for retargeting UniCAR-expressing T cells (UniCAR-Ts) to EGFR-expressing cancer cells [108]. UniCARs do not redirect T cells against a particular TAA or tumor-specific antigen (TSA). Instead, UniCARs are redirected towards a unique peptide epitope on recombinant targeting modules [108]. Therefore, UniCARs can be redirected towards a cell surface antigen of interest in the presence of a targeting module redirected towards that given antigen [108]. Albert et al. reported that their VHH-based EGFR-redirected targeting modules effectively redirected UniCAR-Ts to EGFR-expressing tumors cells [108]. In detail, CAR-Ts redirected using these targeting modules mediated effective target antigen-dependent tumor cell lysis both in vitro (against EGFR-expressing A431 and FaDu cell lines) and in vivo (in preclinical mouse models established using A431 cells) [108]. Albert et al. also added that, in terms of biodistribution, unbound targeting modules were rapidly eliminated [108]. Furthermore, in 2018, Albert et al. also generated a novel bivalent α-EGFR-EGFR targeting module and reported that this bivalent UniCAR-redirecting module had higher avidity in comparison to that of its monovalent counterpart (Fig. 5) [109]. They also added that monovalent EGFR-redirected targeting modules could only induce antitumor activity when they encountered high-level EGFR expression on tumor cells while bivalent α-EGFR-EGFR-redirected targeting modules could meditate UniCAR-T-induced antitumor activity towards cancer cells expressing low levels of EGFR [109]. Based on the in vivo assessments, the increased avidity of the bivalent version of this targeting module enhanced tumor site trafficking and distribution suggesting its superior capability for PET imaging [109]. Overall, it could be concluded that these reports are the first ones on the applicability of monovalent and bivalent VHH-based targeting modules for retargeting UniCAR-Ts against a specific neoplasm-associated target antigen. Alongside novelty, this approach has demonstrated that nanobodies can be used in the construct of UniCAR-T targeting modules, and they can mediate specific redirection of UniCAR-Ts resulting in efficient target tumor cell elimination. It is worth mentioning that further preclinical and clinical investigations can better highlight the applicability of this platform.

#### CD20

CD20 is a transmembrane B-cell marker expressed during B-cell differentiation with important roles in the development and differentiation of B cells into plasma cells [110]. *Rituximab* was the first CD20-specific mAb approved by the FDA in 1998 [110]. Since then, *rituximab* has been used as a therapeutic option for many patients with CD20^+^ B-cell malignancies [110]. The encouraging outcomes of targeting CD20 using *rituximab* made this cell surface marker an interesting target for various types of cancer immunotherapy. CAR-T-mediated targeting of CD20 has been investigated in various phases of clinical trials using CAR-Ts with scFv-based targeting domains [111]. In 2018, De Munter et al. generated a panel of VHH-based CAR-Ts consisting of monomeric VHH-based CD20-redirected and HER2-redirected CAR-Ts and bispecific VHH-based CAR-Ts specific for CD20 and HER2 [112]. According to the findings of this study, the two monomeric CAR-Ts demonstrated target antigen-dependent effector cell activation and expansion, cytokine production, and tumor cell lysis upon co-cultivation with Jurkat cells genetically engineered to express CD20 or HER2 or both on their surface [112]. Moreover, bispecific CAR-Ts also exhibited similar antitumor activity, as their monomeric counterparts, upon co-culture with Jurkat cells engineered expressing either CD20 or HER2 or both antigens simultaneously [112]. Collectively, these researchers proposed that the generation of compact CAR-Ts with dual specificity and predefined affinity can be achieved using nanobody technology [112]. However, this investigation was only a proof-of-concept study on the application of nanobodies for the development of bispecific VHH-based CAR-Ts. Moreover, the discovery of two target antigens that could be targeted using bispecific CAR-Ts for the elimination of tumor cells without mediating any bystander off-tumor effects on healthy tissues is the main limitation of this approach.

Moreover, in 2020, De Munter et al. used DNA vaccination for immunizing llamas against CD20 and generating CD20-specific nanobodies [93]. These researchers used a specific isolated monoclonal nanobody for generating VHH-based CD20-redirected CAR-Ts and evaluating the antitumor activity of these cells in vitro and in vivo [93]. The Burkitt lymphoma cell line Raji, non-Hodgkin B lymphoblast cell line RL, and transgenic Jurkat cells genetically engineered to stably express CD20 were used as CD20-expressing target tumor cells [93]. De Munter et al. reported that their CAR-Ts demonstrated significant target antigen-specific tumor cell lysis and cytokine secretion [93]. Moreover, VHH-based CD20-redirected CAR-Ts mediated complete elimination of subcutaneous tumors in mouse xenograft models (established using CD20-expressing RL cells) and considerably extended the survival of these models [93]. Overall, CD20-redirected scFv-based CAR-Ts are being majorly investigated for the treatment of various types of B-cell based malignancies, especially in patients who have not responded to CD19-rdirected CAR-T therapy [111, 113]. Such data might support the applicability of this target antigen for CAR-T therapy of CD20^+^ hematologic malignancies.

#### PD-L1 and EIIIB

CAR-T therapy is mainly based on targeting cell surface-expressed tumor-associated markers. However, solid tumors do not often express a unique surface antigen marker that can be efficiently targeted using CAR-Ts. Additionally, exploiting neoantigens for cancer therapy may be time-consuming and expensive since this process requires the development of mAbs (more specifically scFvs or VHHs) for generating CAR-Ts redirected against a particulate type of a neoantigen. In this regard, researchers have leveraged certain features of the TME to achieve CAR-T-meditated tumor outgrowth suppression. For instance, inhibitory molecules such as PD-L1 are expressed in many solid tumors [114, 115]. Tumor cells as well as the tumor-infiltrating myeloid cells broadly express PD-L1 [114, 115]. As a result, CAR-T-mediated PD-L1 targeting can tackle immune system suppression and simultaneously lead to selective CAR-T-mediated responses in the TME.

Solid tumors are extremely dependent on the extracellular matrix (ECM) and neovasculature for meeting their essential nutrient demands. Solid tumor ECM and neovasculature express exclusive antigens that are not expressed in healthy tissues [116, 117]. Therefore, this feature of solid tumor tissues can also be leveraged as a basis for CAR-T therapy. One of these targets against which CAR-Ts have been generated is a splice variant of fibronectin named *EIIIB*. EIIIB is robustly expressed by tumor ECM and neovasculature which makes it an ideal target for solid tumor CAR-T therapy [116, 118].

In 2019, Xie et al. generated separate VHH-based CAR-Ts redirected against PD-L1 (to specifically target the TME) and EIIIB (to specifically target the tumor stroma and vasculature) [119]. in vitro and in vivo assessments demonstrated that these CAR-Ts mediated delayed and declined tumor outgrowth and prolonged mouse model survival [119]. Of note, in vivo evaluations were carried out using the fully syngeneic B16 melanoma models and PD-L1-overexpressing B16 melanoma models as well as colon MC38 cell line-established adenocarcinoma model in immunocompetent mice [119]. In detail, the administration of EIIIB-redirected CAR-Ts resulted in efficient tumor infiltration and necrosis [119]. Moreover, these researchers added that targeting tumor stroma and neovasculature helped establish a tumor site inflammatory reaction resulting in sequential immune responses [119]. Xie et al. suggested that since various solid tumors rely on the ECM and neovasculature for survival, EIIIB targeting is not limited to a specific tumor type [119].

In 2020, Xie et al. stepped further by generating VHH-based CAR-Ts that secret VHHs redirected against CD47, PD-L1, or CTLA-4 as well as anti-CD47 VHH-Fc fusion [120]. CD47 is a membrane-spanning protein acting as a *“don't eat me signal”* to phagocytes [121]. The overexpression of CD47 has been observed in various types of malignancies [121]. Preclinical studies have demonstrated that CD47 blockade correlates with enhanced antitumor activity of mAb therapy in different cancer models [122–125]. Xie et al. evaluated the efficacy and antitumor activity of VHH-secreting CAR-Ts in vitro and in vivo [120]. They reported that the secretion of anti-CD47 nanobodies by CAR-Ts resulted in enhanced involvement of the innate immune system, triggered epitope spreading, and improved tumoricidal responses [120]. Furthermore, CD47 blockade therapy can be considered a promising approach if the related toxicities of its systemic application are resolved [126]. In this regard, Xie et al. demonstrated that anti-CD47 VHH-Fc fusion secretion by CAR-Ts in tumor sites led to enhanced tumoricidal activity (in comparison with conventional CAR-T therapy) alongside preventing toxicities correlated with the systemic administration of anti-CD47 VHH-Fc fusions [120]. These researchers also added that localized secretion of nanobodies redirected against PD-L1 or CTLA-4 resulted in enhanced persistence of CAR-Ts [120]. Overall, Xie and colleagues demonstrated that CAR-Ts (more specifically VHH-based CAR-Ts) can be efficiently engineered to secrete nanobodies with immunomodulatory characteristics resulting in improved CAR-T-mediated tumoricidal responses [120]. In conclusion, it can be suggested that selective targeting of the TME and the tumor stroma and vasculature though direct CAR-T-mediated targeting or engineering CAR-Ts to secrete nanobodies specific for the mentioned markers may result in improved innate immune system reactions and antitumor responses in vivo. However, substantiated clinical data are required to elucidate if this approach can amplify antitumor responses through targeting tumor stroma and vasculature without any or with negligible toxicities towards the blood vessels of healthy tissues.

#### CD105

CD105, also known as *endoglin*, is a transmembrane glycoprotein that acts as a co-receptor for transforming growth factor-beta (TGF-β) [127, 128]. CD105 overexpression has been observed in proliferating endothelial cells, and it has been proposed as an ideal marker for neoplasm-associated angiogenesis and neovascularization [127, 128]. The expression level of this marker correlates with reduced patient survival and metastasis in various solid tumors [128]. In 2021, Mo et al. generated CD105-redirected CAR-Ts using a CD105-specific nanobody as the CAR targeting domain [129]. First, these researchers characterized the expression level of CD105 in various hepatocellular carcinoma cell lines, human umbilical vein endothelial cell (HUVEC) line, and 293 T cells [129]. According to their results, Bel7404, HepG2, SMMC7721, and HUVEC cells overexpressed CD105 whereas MHCC97H either had negligible expression and 293 T cells did not have any expression [129]. The co-cultivation of VHH-based CD105-redirected CAR-Ts with CD105-expressing Bel7404 cells in vitro led to the overexpression of T lymphocyte activation markers (including CD25 and CD69) on the surface of CAR-Ts [129]. Moreover, these CAR-Ts demonstrated significant expansion, pro-inflammatory cytokine expression, and specific antitumor activity against CD105-expressing cells in vitro [129]. Additionally, in vivo characterization of these CAR-Ts in Bel7404 cell line-established xenograft mouse models demonstrated that these CAR-Ts mediated tumor outgrowth suppression, reduction in the tumor bulk size, and improved overall survival of the xenograft models [129]. According to these findings, the researchers proposed that CD105-redirected nanobody-based CAR-Ts can have beneficial antitumor activity for the selective targeting of solid tumors [129]. However, since this is the only report on CAR-T-mediated targeting of CD105 to our knowledge, we believe that broader investigations (using conventional scFv-based CAR-Ts as well) can help elucidate the suitability of this target antigen for the treatment of various types of solid tumors.

#### B-cell maturation antigen (BCMA)

BCMA is a membrane-spanning activator and calcium modulator with important roles in the regulation of B-cell maturation and differentiation into plasma cells [130]. The high-level expression of BCMA on malignant plasma cells has rendered it a great target antigen for various types of cancer immunotherapy [131]. BCMA is also an interesting CAR-T therapy target [132]. In 2020, US FDA approved *idecabtagene vicleucel* (also known as *Abecma*) for clinical application making it the first cell-based cancer therapy for the treatment of certain patients with R/R MM [7]. The targeting domain of this CAR-T product is a BCMA-specific scFv [7]. However, BCMA-specific nanobodies have also been utilized as the targeting domain of CAR-Ts.

In 2018, Zhao et al. reported the findings of a Phase I clinical trial (NCT03090659) investigating the safety and efficacy of autologous VHH-based BCMA-redirected CAR-Ts (named *LCAR-B38M*) in patients with R/R MM [133]. *LCAR-B38M *CAR-Ts are bi-epitopic CAR-Ts redirected against two different BCMA epitopes [133]. According to the findings, the adverse events included CRS, which was documented in 51 out of 57 patients (90%) with only 4 patients experiencing severe CRS (grade ≥ 3), pyrexia (in 91% of the patients), thrombocytopenia (in 49% of the patients), and leukopenia (in 47% of the patients) [133]. Coagulopathies were also documented in patients that experienced CRS. Additionally, liver function-related abnormalities, including elevated levels of aspartate aminotransferase (AST), were the principal indicators of end-organ injury among those experiencing CRS [133]. The overall response was reported to be 88% and 39 patients (68%) experienced CR [133]. Moreover, MRD was negative in 36 patients (63%) [133]. Overall, the findings of this ongoing clinical trial demonstrated that *LCAR-B38M* CAR-Ts are well-tolerated and mediate durable clinical responses in R/R MM patients [133]. Moreover, in 2019, Xu et al. published another report of the same clinical trial (NCT03090659) investigating the clinical responses of *LCAR-B38M* CAR-Ts in 17 patients with R/R MM [134]. In terms of adverse events following CAR-T therapy, 10 patients (58.8%) demonstrated mild CRS, 6 patients (35.2%) had severe but controllable CRS, and one patient (5.8%) died due to severe CAR-T infusion-related complications [134]. The overall response rate was reported to be 88.2% with 13 patients (81.2%) experiencing stringent complete response (sCR), 2 patients (12.5%) achieving very good partial response (PR), and 1 patient (6.2%) without any clinical response [134]. These findings further confirmed the promising capability of these bi-epitopic CAR-Ts for the treatment of MM patients with manageable adverse events [134]. Various other clinical trials are currently investigating the applicability of these CAR-Ts for the treatment of R/R MM patients and, so far, similar clinical outcomes have been reported [135–137].

On February 28, 2022, the US FDA approved *ciltacabtagene autoleucel* (also known as *cilta-cel* or *CARVYKTI*) for the treatment of adult patients with R/R MM [12]. *Cilta-cel*, which uses a CAR construct identical to *LCAR-B38M*, is a CAR-T product approved for medical use in the mentioned patients who have been nonresponsive to at least four prior types of other MM therapy approaches including proteasome inhibitor (PI) therapy, immunomodulatory agent therapy, and CD38-specific mAb therapy. *Cilta-cel* has been approved based on the findings of the open-label multicenter clinical trial *CARTITUDE-1* (NCT03548207) in which the safety and efficacy of this CAR-T product were evaluated in 97 adult patients with R/R MM [137]. According to the report by Berdeja et al., the patients received autologous CAR-expressing viable T cells at a dose of 0.5–1.0 × 10^6^/kg body weight [137]. The reported overall response rate was 97% (in 94 of 97 patients), and sCR was documented in 65 patients (67%) [137]. The time to first response was reported to be 1 month. Moreover, it was reported that the clinical responses in patients improved over time [137]. In this regard, the 12-month progression-free rate and overall survival rate were 77% and 89%, respectively [137]. In terms of side effects, the occurrence rate of grade 3–4 hematological adverse events was high with neutropenia in 92 patients (95%), anemia in 66 patients (68%), leukopenia in 59 patients (61%), thrombocytopenia in 58 patients (60%), and lymphopenia in 48 patients (50%) [137]. Moreover, CRS was observed in 92 patients (95%) but only 4% demonstrated grade ≥ 3 CRS [137]. According to this report, CRS was manageable in all of the patients except for one with grade 5 CRS and hemophagocytic lymphohistiocytosis [137]. Also, neurologic toxicities were reported in 20 patients (21%) but only 9% experienced grade ≥ 3 neurotoxicity [137]. Of 97 patients, 14 died due to CAR-T infusion-related side effects, disease progression, or treatment-unrelated adverse events [137]. In a nutshell, these findings demonstrated that *cilta-cel* can mediate immediate, deep, and prolonged clinical responses in R/R MM patients nonresponsive to particular lines of prior therapies [137].

Additionally, in 2019, Han et al. reported the results of a clinical trial (NCT03661554) investigating the safety and efficacy of autologous second-generation BCMA-redirected CAR-Ts, with humanized alpaca-derived anti-BCMA nanobodies as their targeting domains, in patients with R/R MM [56]. In detail, as of December 31, 2018, 16 patients (3 with extramedullary disease and 13 without extramedullary disease) received these CAR-Ts. On day 28, the 3 patients with extramedullary disease achieved PR [56]. Among the 13 patients without extramedullary disease, the overall response rate was reported as 84.6% [56]. In terms of CAR-T infusion-related side effects, only two patients experienced high-grade CRS (grade 3 or 4) and the rest of the patients had mild CRS (grade 0 to 2) [56]. Such results exhibited the efficacy and manageable safety profile of these CAR-Ts in patients with R/R MM [56]. In 2021, Han et al. published another report of the findings of this clinical trial [55]. According to this report, as of February 1, 2021, 34 MM patients were treated, all of which had plasma cell burden in the bone marrow, and in-serum M protein or free light chains [55]. Moreover, 7 patients had extramedullary disease [55]. In terms of efficacy, the overall response rate was 88.2%, sCR was 55.9%, and median progression-free survival (mPFS) was more than one year [55]. The adverse events included neutropenia (44.1%), lymphopenia (26.5%), leukopenia (32.4%), thrombocytopenia (38.2%), and anemia (20.6%), all of which were ≥ grade 3 [55]. Moreover, CRS (of any grade) was experienced by 29 patients (85.3%) [55]. Such findings further highlight the efficacy, as well as the safety, of these humanized nanobody-based CAR-Ts for the treatment of patients with R/R MM.

These CAR-Ts have also been administered to MM patients with chronic or resolved hepatitis B virus (HBV) and similar promising clinical outcomes have been documented (NCT03664661) [54]. In detail, it has been suggested that there is a risk of HBV infection reactivation following CAR-T therapy in R/R MM patients [54]. Han et al. administered autologous nanobody-based BCMA-redirected CAR-Ts to 9 R/R MM patients with chronic or resolved HBV infection [54]. Following CAR-T administration, the patients’ sera were examined to evaluate the expression of different HBV components as well as the presence of HBV DNA [54]. According to the results, no HBV reactivation was reported. However, one patient demonstrated recurrence of hepatitis B surface antigen which was not accompanied by the detection of HBV DNA or liver function abnormalities [54]. In conclusion, these researchers reported that autologous nanobody-based BCMA-redirected CAR-Ts can be employed for the treatment of patients with R/R MM with chronic or resolved HBV infection, and it is recommended to use antiviral drugs in these patients during the course of CAR-T therapy [54]. However, more in-depth clinical outcomes in broader patient populations are required to safely rule out such hypotheses.

## Conclusion

CAR-T therapy represents a specific class of genetically engineered T-cell-based immunotherapeutics that can be feasible, safe, and effective for the treatment of conventional treatment-resistant hematologic neoplasms. However, these “*living drugs*” face multiple challenges in regards to their targeting domains. scFvs, as the most common targeting domain of CARs, tend to have limitations that can appear as obstacles to the safety and efficacy of CAR-T products after administration. The major limitations of scFv-based CAR-Ts include the emergence of anti-idiotypic responses against the CAR targeting domain (due to the presence of the linker peptide or the murine origin of the scFv), and scFv aggregation resulting in pre-mature and antigen-independent CAR-T exhaustion. However, five scFv-based CAR-T products have been approved by the US FDA so far, suggesting that this platform can still be effective and safe for in-human applications and the treatment of patients with drug-resistant hematologic neoplasms (aside from all the mentioned hurdles regarding the application of scFv-based CAR-Ts). As there is always room for improvement, in cases where such limitations may impede the antitumor activity of CAR-Ts and render them dysfunctional, researchers have proposed the application of alternative targeting domains, such as nanobodies, which can resolve the mentioned scFv-based CAR-T limitations to a large extent. As underscored throughout this article, nanobody-based CAR-Ts could be as effective as conventional CAR-Ts with scFv-based targeting domains. Multiple studies have demonstrated that VHH-based CAR-Ts exhibit target antigen-dependent cytotoxicity against various types of malignancies in vitro, in preclinical xenograft models, and in clinical studies. Moreover, nanobodies might not be able to aggregate on the surface of T cells because of their monomeric structure [32]. Therefore, they might be beneficial in terms of preventing premature T cell activation and exhaustion which is independent of target antigen engagement and happens during the process of scFv aggregation [32]. Furthermore, nanobodies do not have the limitation of affinity loss which is recognized as a possible side effect in the design of scFvs [138, 139]. It is worth mentioning that, up until February 2022, all of the FDA-approved CAR-T products were CAR-Ts with scFv-based targeting domains, and *ciltacabtagene autoleucel* was the first VHH-based CAR-T product approved by the US FDA. The encouraging clinical outcomes of this BCMA-redirected nanobody-based CAR-T product in R/R MM patients paved the way for its FDA approval suggesting that nanobody-based CAR-T products can be as effective and well-tolerated as conventional scFv-based CAR-Ts in the clinics and they might be able to mediate disease remission in patients with R/R hematologic malignancies.

## Data Availability

Not applicable.

## Abbreviations

CAR-T: Chimeric Antigen Receptor T-cell
MHC: Major Histocompatibility Complex
R/R: Relapsed/Refractory
B-ALL: B-cell Acute Lymphoblastic Leukemia
FDA: Food and Drug Administration
DLBCL: Diffuse Large B-cell Lymphoma
FL: Follicular Lymphoma
MCL: Mantle Cell Lymphoma
MM: Multiple Myeloma
scFv: Single-Chain Fragment Variable
mAb: Monoclonal Antibody
TME: Tumor Microenvironment
TRBA: T-cell-Redirecting Bispecific Antibody
VHH: Single Variable Domain on a Heavy Chain
HcAbs: Heavy chain-only Antibodies
V_L_: Variable light-chain
V_H_: Variable heavy-chain
HAMA: Human Anti-Mouse Antibody
CAIX: Carbonic Anhydrase IX
CDR: Complementarity-Determining Region
TanCAR: Tandem CAR
VEGFR2: Vascular Endothelial Growth Factor Receptor 2
VEGF: Vascular Endothelial Growth Factor
HNSCC: Head And Neck Squamous Cell Carcinoma
HER2: Human Epidermal Growth Factor Receptor 2
TAG-72: Tumor-Associated Glycoprotein 72
PSMA: Prostate-Specific Membrane Antigen
GPC2: Glypican 2
ACT: Adoptive Cell Therapy
NK: Natural Killer
AML: Acute Myeloid Leukemia
ADCC: Antibody-Dependent Cell-Mediated Cytotoxicity
HPC: Hematopoietic Precursor Cell
T-ALL: T-cell Acute Lymphoblastic Leukemia
ALL/LBL: Acute Lymphoblastic Leukemia/Lymphoma
ETP: Early T-cell Precursor
CR: Complete Remission
CRS: Cytokine Release Syndrome
MRD: Minimal Residual Disease
MUC1: Mucin 1
EGFR: Epidermal Growth Factor Receptor
TAA: Tumor-Associated Antigen
UniCAR-T: UniCAR-expressing T cell
TSA: Tumor-Specific Antigen
ECM: Extracellular Matrix
TGF-β: Transforming Growth Factor-Beta
HUVEC: Human Umbilical Vein Endothelial Cell
BCMA: B-Cell Maturation Antigen
AST: Aspartate Aminotransferase
sCR: Stringent Complete Response
PR: Partial Response
PI: Proteasome Inhibitor
mPFS: Median Progression-Free Survival
HBV: Hepatitis B Virus
